# Severe Hemoptysis Associated With Probable Tracheobronchial Leech Infestation Following Spontaneous Expectoration: A Case Report

**DOI:** 10.7759/cureus.114750

**Published:** 2026-08-18

**Authors:** Albatoul Debbagh, Youssra El Amrani, Meryem Akassisse, Halloumi Oussama, Hind Serhane

**Affiliations:** 1 Pulmonology Department, Mohamed VI University Hospital, Agadir, MAR; 2 LARISS (Laboratoire de Recherche en Ingénierie des Sciences de la Santé) Laboratory, Faculty of Medicine and Pharmacy of Agadir, Ibn Zohr University, Agadir, MAR

**Keywords:** airway leech infestation, alveolar hemorrhage, bronchoscopy, hirudiniasis, severe hemoptysis, spontaneous expectoration

## Abstract

Airway leech infestation is a rare but potentially life-threatening cause of severe hemoptysis, usually occurring after exposure to untreated freshwater in rural or endemic areas. Mechanical mucosal injury, together with the anticoagulant and antiplatelet effects of leech saliva, including hirudin and other bioactive substances, may contribute to persistent bleeding, while nonspecific symptoms can delay diagnosis. We report the case of a 56-year-old chronic smoker who was admitted with severe hemoptysis, dysphonia, and mild dyspnea after recent exposure to untreated freshwater. Computed tomography pulmonary angiography showed parenchymal consolidations associated with bilateral ground-glass opacities suggestive of alveolar hemorrhage, without pulmonary embolism, vascular malformation, or structural bronchial abnormality. On the second day of hospitalization, the patient spontaneously expectorated a live leech measuring approximately 5 cm in length. This was immediately followed by complete cessation of hemoptysis and rapid clinical improvement. Flexible bronchoscopy performed after expectoration showed a normal tracheobronchial tree, with no active bleeding, residual parasite, endobronchial lesion, or mucosal abnormality. No recurrence was observed during a three-month follow-up period. This case highlights the need to consider airway leech infestation in patients with unexplained severe hemoptysis, particularly in endemic regions or after exposure to untreated freshwater. Bronchoscopy is the standard diagnostic and therapeutic approach in most reported cases, although spontaneous expectoration may rarely result in complete recovery.

## Introduction

Severe hemoptysis is a medical emergency associated with significant morbidity and mortality if not promptly recognized and managed [1]. Infectious, neoplastic, and vascular diseases account for most cases; however, rare etiologies, such as parasitic infestation, should also be considered in the appropriate epidemiological context. Leech infestation, or hirudiniasis, is an uncommon but documented cause of respiratory bleeding, typically occurring after exposure to untreated freshwater in endemic rural areas. Human internal hirudiniasis may develop after accidental ingestion of contaminated water or direct exposure while bathing or swimming. After entering through the oral or nasal route, the leech may attach to the mucosa of the nasopharynx, oropharynx, or larynx and, less commonly, reach the tracheobronchial tree, where it adheres to the well-vascularized mucosa and feeds on blood [2]. Respiratory manifestations therefore depend on the site of attachment and may range from dysphonia or throat discomfort to cough, dyspnea, hemoptysis, or, in severe cases, airway obstruction.

Leeches are hematophagous annelids that attach to mucosal surfaces using suckers and feed by penetrating the mucosa. This attachment causes direct mechanical mucosal injury. They also secrete saliva containing biologically active substances, including hirudin, a potent thrombin inhibitor. The combination of mechanical mucosal injury and the anticoagulant and antiplatelet effects of leech saliva may contribute to prolonged bleeding. Their local anesthetic effects can also delay recognition of the infestation [3].

Because clinical manifestations are nonspecific and may include cough, hemoptysis, dyspnea, or dysphonia, diagnosis can be delayed or overlooked. Awareness of this entity is therefore important, especially in rural or endemic settings. We report the case of a 56-year-old chronic smoker admitted for severe hemoptysis associated with probable airway leech infestation, in whom spontaneous expectoration of the parasite resulted in complete clinical recovery. This case illustrates an uncommon clinical scenario combining spontaneous expectoration of an airway leech before bronchoscopic evaluation with radiological findings suggestive of alveolar hemorrhage.

## Case presentation

A 56-year-old male manual laborer and chronic smoker with a 30 pack-year smoking history and no history of tuberculosis or recent infectious exposure was admitted for severe hemoptysis. He reported recent exposure to untreated freshwater in a rural area approximately two weeks before symptom onset.

Symptoms began nine days before admission with productive cough and blood-streaked sputum, initially without chest pain or persistent dyspnea. The clinical course progressively worsened, with recurrent large-volume hemoptysis estimated at approximately 200-300 mL per episode. The exact cumulative 24-hour volume was not documented. Bleeding episodes were associated with dysphonia and mild dyspnea. Reported hematemesis was considered likely secondary to swallowed blood. No fever, weight loss, night sweats, or other systemic symptoms were reported.

On admission, the patient was alert and fully oriented, with a Glasgow Coma Scale score of 15/15. Physical examination revealed mild mucocutaneous pallor without cyanosis, peripheral edema, or digital clubbing. Vital signs were stable: respiratory rate 18 breaths/min, oxygen saturation 97% on room air, blood pressure 105/57 mmHg, and heart rate 93 beats/min.

Laboratory findings on admission are summarized in Table 1. They showed mild anemia, with a hemoglobin level of 12.3 g/dL, and a markedly elevated C-reactive protein level of 205 mg/L. Antineutrophil cytoplasmic antibodies were negative. Blood, urine, and sputum cultures were negative.

**Table 1:** Laboratory findings on admission CRP: C-reactive protein; ANCA: antineutrophil cytoplasmic antibodies Reference ranges may vary according to local laboratory standards.

Laboratory parameter	Result	Reference range	Interpretation
Hemoglobin	12.3 g/dL	13.0-17.0 g/dL	Mild anemia
C-reactive protein	205 mg/L	<5 mg/L	Elevated
Antineutrophil cytoplasmic antibodies	Negative	Negative	Normal
Blood cultures	Negative	Negative	Normal
Urine culture	Negative	Negative	Normal
Sputum culture	Negative	Negative	Normal

Computed tomography pulmonary angiography revealed two parenchymal consolidations associated with bilateral ground-glass opacities, suggestive of alveolar hemorrhage. No pulmonary embolism, vascular malformation, or structural bronchial abnormality was identified.

**Figure 1:**
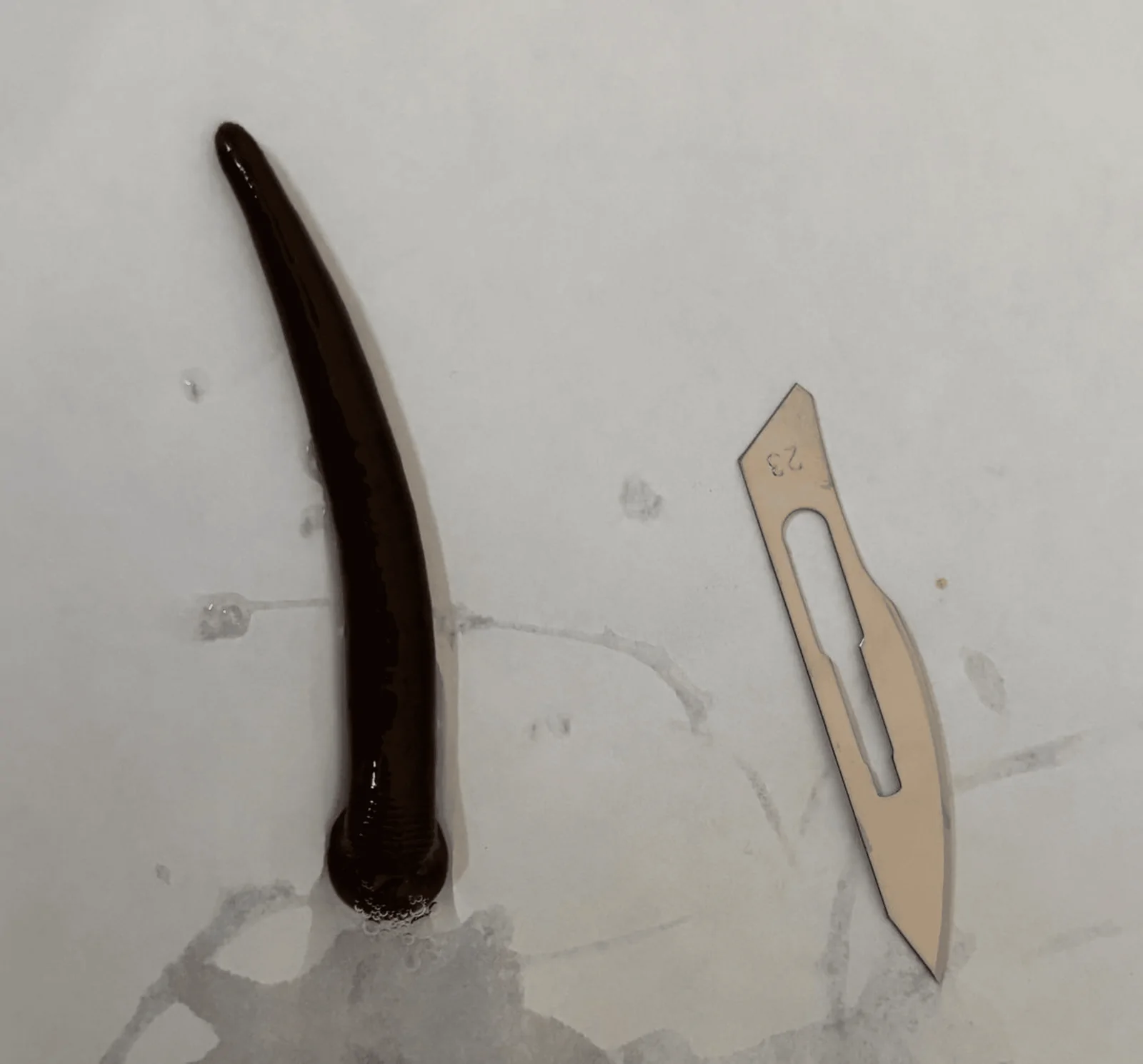
Live leech spontaneously expectorated by the patient, measuring approximately 5 cm in length, placed next to a No. 23 surgical blade for size comparison.

The patient was admitted for close respiratory and hemodynamic monitoring while etiological evaluation was ongoing. No blood transfusion, antibiotic therapy, or specific hemostatic treatment was administered during hospitalization. No inhaled or nebulized therapy, including saline nebulization, was administered before spontaneous expectoration of the leech. On the second day of hospitalization, he spontaneously expectorated a live leech measuring approximately 5 cm in length. This was immediately followed by complete cessation of hemoptysis and rapid clinical improvement. Because the leech was not visualized before spontaneous expectoration, the precise anatomical site of attachment could not be confirmed (Figure 1).

**Figure 2:**
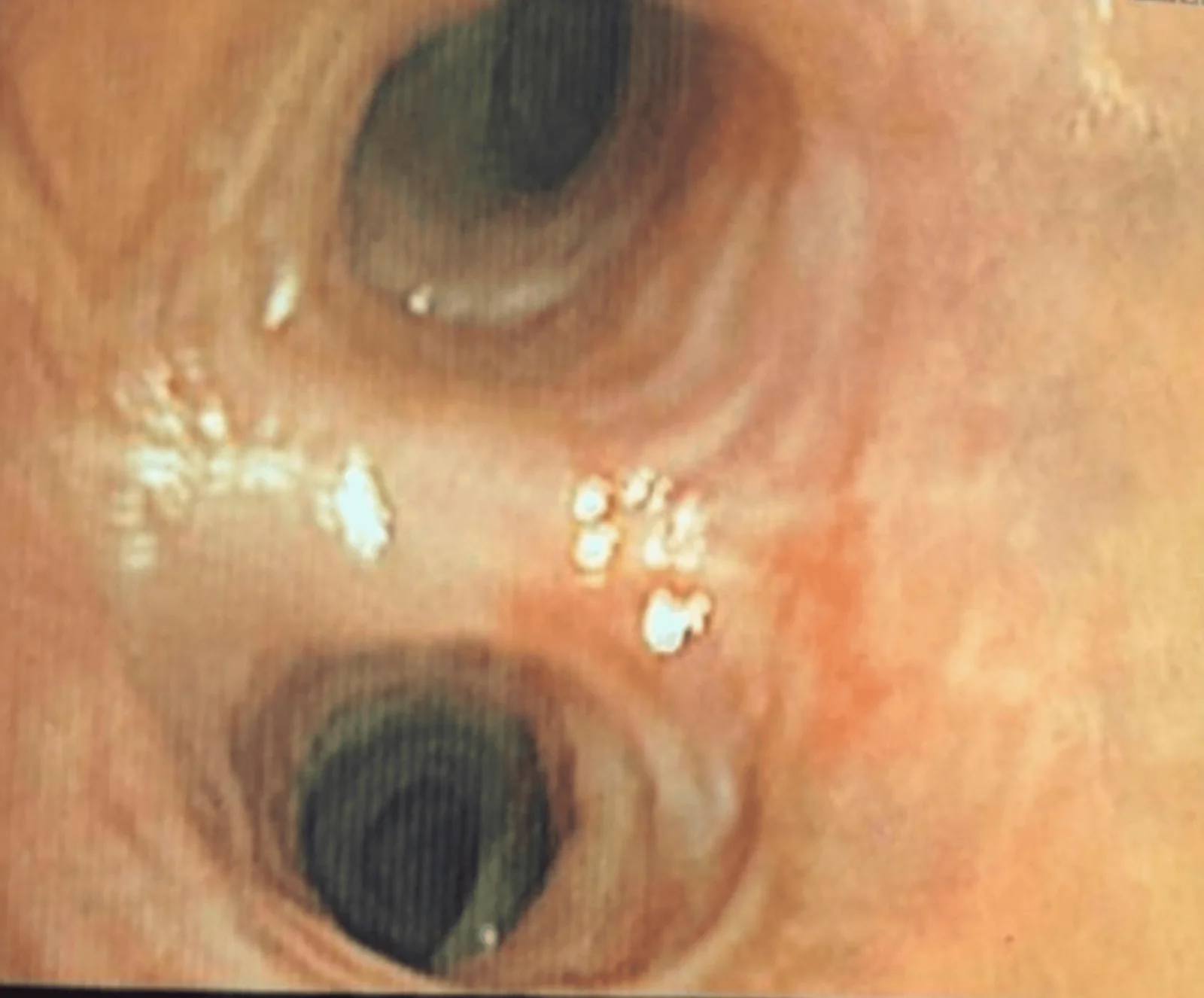
Flexible bronchoscopy performed after spontaneous expectoration of the leech, showing a normal tracheal carina and patent main bronchi, with no active bleeding, residual parasite, or endobronchial lesion.

Flexible bronchoscopy was subsequently performed to assess the airway and rule out persistent bleeding or residual lesions. It demonstrated a macroscopically normal tracheobronchial tree, with a normal tracheal carina and patent main bronchi. No active bleeding, endobronchial lesion, residual parasite, or mucosal abnormality was observed (Figure 2).

Inflammatory markers progressively decreased during hospitalization, and hemoglobin levels improved. No recurrence of hemoptysis was observed during a three-month follow-up period.

## Discussion

Internal hirudiniasis is a rare parasitic infestation caused by leeches, hematophagous annelids belonging to the class Hirudinea. Most species inhabit freshwater environments, although terrestrial and marine species also exist [4]. In human infestation, leeches attach to mucosal surfaces and feed on blood, causing local trauma and prolonged bleeding.

The clinical presentation depends on the anatomical site of attachment. Nasal or nasopharyngeal involvement may cause epistaxis, nasal obstruction, or foreign body sensation. Oral and oropharyngeal infestation may present with dysphagia or odynophagia. Laryngeal involvement can lead to hoarseness or airway obstruction, whereas tracheal or bronchial localization more commonly causes cough, dyspnea, and hemoptysis [5]. In our patient, dysphonia may suggest transient laryngeal or proximal tracheal attachment before detachment and expectoration. A recent case report of nasopharyngeal leech infestation further illustrates the variability of clinical manifestations according to the site of attachment and reinforces the importance of a history of exposure to potentially contaminated freshwater [6].

The clinical findings are consistent with a proximal airway source, although the precise anatomical site of attachment cannot be determined because the parasite was not visualized before expectoration.

The severity and persistence of bleeding may result from both mechanical mucosal injury and the anticoagulant and antiplatelet effects of leech saliva. Hirudin and other anticoagulant and antiplatelet substances may inhibit thrombin activity, impair platelet activation and aggregation, and promote ongoing bleeding even after detachment [7]. Reported manifestations include hemoptysis, epistaxis, hematemesis, and anemia in prolonged cases.

Computed tomography demonstrated bilateral ground-glass opacities and consolidations compatible with alveolar hemorrhage; however, these imaging findings are not specific and should be interpreted in the clinical context [8]. In this case, these findings may have reflected intra-alveolar blood related to distal aspiration of blood and the anticoagulant effects of leech saliva. The markedly elevated C-reactive protein level was nonspecific and may have reflected airway inflammation and blood aspiration in the absence of microbiological evidence of infection.

The differential diagnosis of severe hemoptysis included infectious, neoplastic, vascular, and autoimmune etiologies. Pulmonary tuberculosis, malignancy, pulmonary embolism, vascular malformation, and systemic vasculitis were considered and were reasonably excluded based on the clinical presentation, negative microbiological tests, negative antineutrophil cytoplasmic antibodies, and computed tomography findings. The temporal association between spontaneous expectoration of the leech and the immediate and sustained resolution of bleeding strongly suggests that the parasite was the most likely source of bleeding; however, this relationship cannot be definitively established from a single case.

Bronchoscopy remains the standard diagnostic and therapeutic approach in most reported cases of suspected airway leech infestation. When intervention is required, removal is typically performed endoscopically, either with forceps or suction-assisted extraction, while securing the airway and controlling bleeding. In our patient, bronchoscopy was performed after spontaneous expectoration and showed no residual parasite, mucosal lesion, or active bleeding, suggesting complete detachment with minimal residual injury.

Spontaneous expectoration of an airway leech is uncommon. This case is unusual because it combines severe hemoptysis, radiological findings suggestive of alveolar hemorrhage, and spontaneous expectoration without endoscopic extraction. Environmental exposure history should be considered in patients with unexplained hemoptysis who have epidemiological risk factors, particularly freshwater exposure in endemic regions.

## Conclusions

Probable airway leech infestation is a rare but potentially severe cause of hemoptysis that may mimic more common pulmonary diseases. Mechanical mucosal injury and the anticoagulant and antiplatelet effects of leech saliva may contribute to significant bleeding. Radiological findings, such as bilateral ground-glass opacities and consolidations, may be compatible with alveolar hemorrhage but are nonspecific and should be interpreted in the clinical context. Environmental exposure history should be considered in patients with unexplained hemoptysis who have relevant epidemiological risk factors, particularly freshwater exposure in endemic regions. Early recognition may facilitate timely diagnosis and appropriate management. In rare cases, spontaneous expectoration may be followed by rapid clinical improvement; however, bronchoscopic evaluation remains important to exclude residual infestation or airway injury.
